# Factors related to well-being and psychological health in nursing students: A cross-sectional survey

**DOI:** 10.1016/j.ijnsa.2025.100421

**Published:** 2025-09-10

**Authors:** Beatriz Calderón-Cruz, Uxía García-Sánchez, Jacinto Luis González-Oya, Ángela Prieto-Campo

**Affiliations:** a Statistics and Methodology Unit, Galicia Sur Health Research Institute (IIS Galicia Sur), SERGAS-UVIGO, Vigo, Spain; b Meixoeiro University School of Nursing, SERGAS-UVIGO, Vigo, Spain; c Área Sanitaria de Vigo, Sergas, Vigo, Spain

**Keywords:** Psychological well-being, Psychological health, Nursing students, Lifestyle

## Abstract

**Background:**

Psychological well-being is an important topic in mental health. It is known that university students have a high prevalence of stress and psychological distress, especially students in health sciences fields such as nursing. Despite the growing emergence of this public health issue, research on the factors affecting the mental health of nursing students is limited.

**Objective:**

To estimate the global psychological health and well-being of Spanish nursing students and its associations with sociodemographic and lifestyle factors.

**Methods:**

A cross-sectional study was conducted among nursing students. Descriptive analysis, bivariate analysis, and binary regression models were performed.

**Results:**

A total of 235 students participated, of which 49.4 % had poor psychological well-being and 51.5 % reported high levels of detrimental emotional symptoms. The main factors related to these unfavorable outcomes were smoking (OR > 5, 95 % C.I. 2–16.30), frequent consumption of fast food (OR > 5, 95 % C.I. 2–16.30), sleeping <7 h (OR > 2.5, 95 % C.I. 1.46–5.09), and being in the third year of the University study program (OR 2.13, 95 % C.I. 1.051–4.331).

**Conclusions:**

These results highlight healthy lifestyle habits as the main factors related to psychological well-being. Additionally, they underscore the need to create psychological support strategies for nursing students.

**What is already known**

• Some lifestyle habits and being in the final year contribute to psychological distress.

• Mind-body exercises reduce stress and anxiety in nursing students.

Alt-text: Unlabelled box

**What this paper adds**

• First study in Galicia to assess well-being and mental health in nursing students.

• Nursing students manifest a high prevalence of poor psychological well-being.

• Our findings highlight the need for psychological support and prevention strategies.

Alt-text: Unlabelled box

## Introduction

Mental health and psychological well-being are essential components of individuals' quality of life. These aspects encompass the emotional, cognitive, and behavioral states of human beings. Mental health and psychological well-being reflect a person’s ability to face the daily life challenges, maintain healthy relationships, feel fulfilled, and reach their full potential (Ruggeri et al., 2020).

Mental disorders represent significant disruptions in cognition. Furthermore, emotional and behavioral regulation, are some of the leading causes of morbidity in primary healthcare settings (Purtle et al., 2020; VizHub, 2021). Mental health and related issues are a significant public health concern, yet they have received limited attention and support from healthcare facilities and systems. Governments have implemented insufficient measures to properly address citizens' mental health challenges, hindering efforts to achieve optimal well-being (Zhou et al., 2018). This issue impacts various sectors of society, where undergraduate students stand out among the most affected (Alshammari et al., 2022).

The University stage is a critical period for personal and life development. Various factors interact and play an important role in students' mental health. This complex interplay of variables can be meaningfully interpreted through the conceptual framework of the biopsychosocial model. This model provides a comprehensive lens to understand health as the result of interactions between biological, psychological, and social domains. According to this conceptual framework, biological factors include eating habits, sleep duration, and physical activity, which are key determinants of mental well-being. Psychological factors involve aspects such as independence, emotional regulation, and the capacity for adaptation in the face of academic and personal demands. Meanwhile, social factors comprise work and family responsibilities, as well as environmental changes that accompany the transition to university life (Bolton, 2023).

Findings from the WHO World Mental Health Surveys International College Student Project, indicate that 31.4 % of university students experience a mental disorder during their first academic year, with severe impairment occurring in 20.4 % of the cases. Although, these findings are general, the prevalence varies depending on the university career (Alonso et al., 2018).

Psychological distress is more prevalent among students in health and social care fields compared to those in other academic disciplines and to the general population (Almansoof et al., 2024). Specifically, the prevalence of depressive symptoms in nursing students typically ranges from 21 % to 43 % (Reverté-Villarroya et al., 2021; Tung et al., 2018). This symptomatology can significantly impact concentration, memory, and decision-making abilities, which in turn affects the academic performance of the students. Furthermore, the stressful environment and emotional demands inherent in nursing studies can exacerbate these problems, creating a feedback cycle that perpetuates psychological distress and impacts overall well-being (Campbell et al., 2022; Sánchez Casado and Benítez Sánchez, 2021; Zhou et al., 2023).

Entering a nursing program requires rigorous preparation, as it is a highly competitive and in-demand field. Once enrolled, nursing students face ongoing psychological stress, compounded by intense time pressures and personal life challenges, all of which significantly impact their mental health. Additionally, academic demands are very high, especially in the final years, when students must balance clinical practice with theoretical coursework, simulations, and the final degree project (Chover-Sierra et al., 2024).

In Spain, the undergraduate nursing program spans four years (BOE-A-2008-12388 Orden CIN/2134/2008, 2008; “Estudios - Escuela Universitaria de Enfermeria Miexoeiro”, n.d.). Typically, the first year focuses on theoretical coursework, with the beginning of clinical practice in the second year and the intensification of these clinical practices in the third year. In the fourth and final year, students must balance their academic workload with the undergraduate thesis, which must be defended before a university committee to obtain the degree.

Although, some studies (Arias-De la Torre et al., 2019; Palenzuela-Luis et al., 2023; Tiga-Loza et al., 2024) have previously employed the General Health Questionnaire (GHQ-12) to assess mental health in Spanish nursing students - reporting that >50 % of participants, especially women and second-year students, may experience psychological distress - there remains a notable gap in the literature. To our knowledge, no published research to date has simultaneously evaluated both general mental health using the GHQ-12 and psychological well-being through the Psychological General Well-Being Index (PGWBI) in this population.

This gap is particularly significant given the increasing academic, emotional, and clinical demands faced by nursing students, which may impact their psychological well-being in nuanced ways not fully captured by a single assessment tool. By integrating both the GHQ-12 and PGWBI, our study offers a more comprehensive evaluation of mental health, encompassing both distress and well-being dimensions.

Moreover, few existing studies have thoroughly examined how psychological health in Spanish nursing students relates to sociodemographic variables (such as age, gender, and academic year) and lifestyle factors (such as physical activity, sleep, or social support). These factors are crucial to understanding vulnerability and resilience patterns in this high-risk group (Palenzuela-Luis et al., 2023; Ramón-Arbués et al., 2023).

In this context, the present cross-sectional study addresses a critical gap by evaluating the global psychological health and well-being of Spanish nursing students using a dual-instrument approach. It also explores the associations between psychological outcomes and sociodemographic and lifestyle variables. Our findings aim to inform the development of targeted strategies to promote mental health and support well-being in nursing education.

## Materials and methods

### Study design and population

This cross-sectional analytical study was carried out through an anonymous survey applied to students enrolled in one of the four nursing schools in southern Galicia, affiliated with the University of Vigo. The participating schools included Pontevedra, Ourense, Meixoeiro, and Povisa.

### Procedure

In February 2023, an invitation to participate in the study was sent via email to students through the academic and information technology (IT) departments of the schools. This invitation contained a link to an online questionnaire on general psychological health and well-being. The survey remained open for one month and was closed in March 2023. All questions were mandatory, meaning that respondents had to answer every question in order to submit the survey. This ensured that no data were missing. Due to the study design and the anonymization of collected information, participants were not required to sign informed consent.

### Instruments used

The questionnaire consisted of 55 questions with Likert-type and multiple-choice response options.

Twenty-two questions corresponded to a PGWBI validated in the Spanish population (Badia et al., 1996). This index provides a global score for the evaluation of the emotional state of each individua. Also, it groups its 22 items into six dimensions: Anxiety, depression, well-being, vitality, self-control, and general health. Each item is rated on a six-point Likert scale to indicate the degree of intensity or frequency experienced in the last week. The global score can range from 22 (severe distress) to 132 points (positive well-being). The six dimensions are interpreted similarly, lower scores indicate lower psychological well-being and higher scores indicate greater well-being. The scale range for each dimension varies depending on the number of items that comprise it.

Another twelve questions were related to a GHQ-12 validated for the Spanish population (Sánchez López and Dresch, 2008). This self-administered instrument is designed to detect psychological morbidity and potential cases of non-psychotic psychiatric disorders, both in primary care patients and in the general population. A global score is calculated from the responses, and according to Rocha et al. (2011), the 12 items can also be grouped into three factors: Coping strategies, self-esteem, and stress. Each item is answered on a four-point Likert scale. All scores are interpreted the same, higher punctuations indicate a greater degree of emotional symptomatology (Sánchez López and Dresch, 2008).

To assess lifestyle behaviors, six questions on physical activity, sleep habits, alcohol consumption, tobacco use, number of meals per day, and type of food consumed were asked to the students. These were the six common health-related activities/habits reported by various studies (Breslow and Enstrom, 1980; Leyton et al., 2018; Rocha KB et al., 2011.).

The remaining questions included sociodemographic (sex, age, year of study, etc.) and individual situations (whether if nursing was their first study choice, if they like the career, etc.).

### Sample size

To calculate the sample size, we considered as a finite population, the number of students enrolled in the participating nursing schools, in the year in which the survey was conducted (777 students). Thus, we calculated the sample size using the formula for estimating the mean of a continuous variable (PWBI Score). We assumed a standard deviation of 21, with a 95 % bilateral confidence interval and a precision of 2.24, yielding an estimated sample size of 235 participants.

Calculations were performed using Ene 3.0 software, developed by the Applied Statistics Service of the Autonomous University of Barcelona and distributed by GlaxoSmithKline laboratories.

### Statistical analysis

We carried out a descriptive analysis, where qualitative variables were presented as percentages and absolute frequencies. Since no quantitative variable had a normal distribution, they were reported as median and interquartile range. We used the Kolmogorov-Smirnov test to assess normality.

To estimate the direct relationship between each index and the sociodemographic, lifestyle, and individual variables, we conducted a bivariate analysis using nonparametric tests, as none of the study variables followed a normal distribution. For comparisons between indices and dichotomous categorical variables, we applied the Mann-Whitney U test; for polytomous variables, we used the Kruskal-Wallis test; and for quantitative variables, we employed Spearman’s correlation. This test was also used to correlate the global results of the two indices under study.

We dichotomized the global PGWBI and GHQ-12 scores, using the median of each as a cutoff. We then conducted a bivariate analysis with all study variables, applying chi-square and Mann-Whitney U tests according to the type of variable. Finally, we developed two binary logistic regression models, one for PGWBI and one for GHQ-12, these models included only factors with statistically significant results in the bivariate analysis.

Analyses were conducted with a 95 % confidence level using IBM’s Statistical Package for Social Sciences (SPSS) version 29 in Spanish.

### Ethical and legal aspects

This study was conducted in compliance with current Spanish legislation and regulations on data protection and biomedical research, ensuring the ethical and scientific standards outlined in these laws and in accordance with the Principles of the Declaration of Helsinki and the Oviedo Convention. The study was reviewed and approved by the Research Ethics Committee of Pontevedra-Vigo-Ourense with registration code 2022/463. Participants were not required to sign informed consent due to the anonymous nature of the study.

## Results

The survey was distributed to a total of 777 students enrolled in the schools, and 235 responded, resulting in a response rate of 30.2 %; sociodemographic and lifestyle characteristics are shown in Table 1. More women than men participated (86.8 % vs. 12.8 %, respectively), although, the percentage of participants per academic year was balanced (First year 30.2 %, second year 23.8 %, third year 22.1 %, and fourth year 23.8 %). The median age was 21 years, and 95.3 % were Spanish nationals.

**Table 1:** Sociodemographic and lifestyle characteristics of the study population.

Characteristics	Total population *N* = 235 (100 %)
	M (IQR) /*n* (%)
Age	21 (19–23)
Body mass index	22.6 (20.3–25.4)
**Sex**	
Woman	204 (86.8 %)
Men	30 (12.8 %)
Other	1 (0.4 %)
**Country of birth**	
Spain	224 (95.3 %)
Other	11 (4.7 %)
In a relationship	112 (47.7 %)
Children	12 (5.1 %)
**Sexual orientation**	
Heterosexual	176 (74.9 %)
Homosexual	12 (5.1 %)
Bisexual	45 (19.1 %)
Other	2 (0.9 %)
**Housing**	
Roommates	79 (33.6 %)
Family/couple	142 (60.4 %)
Alone	14 (6 %)
**Academic year**	
1°	71 (30.2 %)
2°	56 (23.8 %)
3°	52 (22.1 %)
4°	56 (23.8 %)
Nursing as first option of study	164 (69.8 %)
Satisfied with the nursing career	224 (95.3 %)
Work and study simultaneously	42 (17.9 %)
Regular excersice	157 (66.8 %)
< 7 h of sleep	127 (54 %)
Smoke	32 (13.6 %)
Eat at least 3 meals a day	212 (90.2 %)
**Type of food**	
Homemade cand balanced	190 (80.9 %)
Fast food	45 (19.1 %)
Risky alcohol consumption^1^	20 (8.5 %)

M (IQR): Median (Interquartile range).

1 We have considered risk consumption to intake > 2 Units of Standard Drink (USD)/day in men and > 1 USD/day in women, as indicated by the Spanish Ministry of Health (“Límites de Consumo de Bajo Riesgo de Alcohol”, 2020).

Over 50 % of the study population identified as heterosexual, single, and reported living with their family. Additionally, ninety-eight per cent of participants indicated that nursing was their first study choice, and 95.3 % expressed satisfaction with their career. Regarding lifestyle, most participants reported healthy habits; over 65 % engaged in regular exercise, did not smoke, had at least three meals per day, and considered their diet to be generally balanced and homemade. However, 54 % reported sleeping less than seven hours per night (see Table 1).

We conducted an analysis to identify characteristics associated with low PGWBI scores and its six dimensions, aiming to detect factors linked to poor general psychological well-being. Table 2 shows characteristics with statistically significant results.

**Table 2:** Median Scores of PGWBI and their six dimensions according to sociodemographic and lifestyle characteristics (Only statistically significant results).

Characteristics	Positive Well-being		Vitality		PGWBI Global		Anxiety		Self-control		General health		Depressed mood	
	Cronbach’s α / M (IQR)	p	Cronbach’s α / M (IQR)	p	Cronbach’s α / M (IQR)	p	Cronbach’s α / M (IQR)	p	Cronbach’s α / M (IQR)	p	Cronbach’s α / M (IQR)	p	Cronbach’s α / M (IQR)	p
**Internal consistency**	0.85	–	0.88	–	0.96	–	0.93	–	0.78	–	0.70	–	0.88	–
**Total population**	10 (8–13)	–	10 (7–14)	–	69 (53–83)	–	16 (11–20)	–	10 (7–12)	–	11 (8–13)	–	12 (10–14)	–
**Sleep hours**														
< 7 h	59 (46–77)	<0.00	14 (8–18)	<0.00	12 (10–13)	<0.00	9 (7–11)	<0.00	9 (6–11)	<0.00	10 (7–12)	<0.00	8 (6–11)	<0.001
≥ 7 h	78 (63–91.8)	1	18 (13–22)	1	13 (11–14)	1	12 (9–15)	1	11 (8.3–13)	1	12 (9.3–13)	1	12.5 (10–16)	
**Smoke**														
Yes	52.5 (42–60)	<0.00	10.5 (7–14)	<0.00	11.5 (8.5–12)	<0.01	8 (6.3–10)	<0.01	7 (5–9)	<0.00	8 (7–11)	<0.01	7 (5–9.8)	<0.001
No	72 (55–85)	1	17 (12–20)	1	12 (11–14)	0	11 (8–14)	0	10 (8–12)	1	11 (8–13)	0	11 (8–14)	
**Type of food consumed**														
Homemade and balanced	72 (56–86)	<0.00	17 (12–20)	<0.00	12 (11–14)	<0.00	11 (8–14)	<0.00	10 (8–12)	<0.00	11 (9–13)	<0.00	11 (8–14)	<0.001
Fast food	50 (33–71)	1	9 (6–16)	1	9 (5–12.5)	1	7 (4–10)	1	7 (4–11)	1	8 (6–11)	1	7 (5–10)	
**Studying and working**														
Yes	8.5 (6–11)	0.013	7 (5–13)	<0.01	56 (41.5–73.8)	<0.01	12.5 (7–17)	<0.01	8.5 (5–11)	0.02	8 (6–11.3)	<0.010		
No	10 (8–13)		11 (8–14)	0	71 (55–85)	0	17 (11–20)	0	10 (8–12)	1	11 (8–13)			
**Regular exercise**														
Yes	11 (8–14)	<0.01	11 (8–14)	0.01	72 (55–84)	0.024								
No	9 (6–12)	0	9.5 (5.8–13)	2	63 (44.5–80.8)									
**Academic Year**														
1°	10 (8–13)		11 (8–15)											
2°	11 (8–14)	0.049	11 (9–14)	0.013										
3°	8.5 (5.3–12)		9 (5–12.5)											
4°	10.5 (8–12.8)		10 (7–12.8)											
**Lives with family/partner**														
Yes	10 (8–13.3)	0.041												
No	9 (7–12)													
**Satisfaction with nursing career**														
Yes	10 (13–8)	0.027												
No	7 (5–12)													
**Consumption of at least 3 meals per day**														
Yes	10 (8–13.3)	<0.010												
No	8 (6–10)													

M (IQR): Median (Interquartile range).

We found that low scores in both, the global PGWBI and its 6 dimensions, were associated with sleeping less than seven hours, studying and working simultaneously, smoking, and primarily consuming fast food (*p*< 0.025). Furthermore, all factors included in Table 2 were related to unfavorable results in the “positive well-being” dimension. Additionally, the absence of regular exercise was associated with low scores in the global PGWBI and the “vitality” dimension. Low scores of this later dimension were also associated with being in the third year of the degree.

Regarding the GHQ-12 questionnaire, we performed an analysis to identify characteristics associated with high scores, thus identifying variables related to psychological morbidity. Table 3 shows six characteristics that yielded statistically significant results: studying and working simultaneously, being in the third year of the degree, not exercising regularly, sleeping less than seven hours, smoking, and primarily consuming fast food, all of them were related to high scores in the global GHQ-12 index (*p*< 0.03). Of these six characteristics, all lifestyle factors (studying and working simultaneously, not exercising regularly, sleeping <7 h, smoking, and primarily consuming fast food) were associated with unfavorable results in the “coping strategies” dimension (*p*< 0.010). Likewise, all characteristics in Table 3, except regular exercise, were associated with high scores in the “self-esteem” and “stress” dimensions (*p*< 0.05).

**Table 3:** Median Scores of GHQ-12 index and their three factors according to sociodemographic and lifestyle characteristics (Only statistically significant results).

Characteristics	GHQ-12 Global		Self-esteem		Stress		Coping strategies	
	Cronbach’s α / M (IQR)	p	Cronbach’s α / M (IQR)	p	Cronbach’s α / M (IQR)	p	Cronbach’s α / M (IQR)	p
**Internal consistency**	0.93	–	0.88	–	0.84	–	0.87	–
**Total population**	11 (7–17)	–	3 (1–6)	–	3 (1–5)	–	7 (6–9)	–
**Study and work**								
Yes	15.50 (9–22)	0.010	4 (2–7.3)	<0.01	3.5 (2–6)	0.016	8 (6–11)	0.027
No	11 (7–16)		3 (1–5)	0	3 (1–4)		6 (6–8)	
**Sleep hours**								
< 7 h	14 (10–21)	<0.001	4 (2–6)	<0.001	4 (2–6)	<0.001	8 (6–11)	<0.001
≥ 7 h	9 (6–14)		2 (0–4)		2 (0–4)		6 (5.3–7)	
**Smoke**								
Yes	18.5 (13–23)	<0.001	6 (3.3–8)	<0.001	5 (3–6)	<0.001	10 (6–11)	<0.010
No	11 (7–15)		3 (1–4)		3 (1–4)		6 (6–8)	
**Type of food**								
Homemade cand balanced	10.5 (7–15)	<0.001	3 (1–5)	<0.001	3 (1–4)	<0.001	6 (6–8)	<0.001
Fast food	16 (11–24.5)		5 (3–8.5)		5 (3–7)		9 (6–12.5)	
**Academic Year**								
1°	10 (7–15)		2 (1–4)		3 (0–4)	0.040		
2°	10 (7–16)	0.041	2 (1–5)	0.028	3 (1–5)			
3°	14 (10–21)		4 (2.3–6.8)		4 (2–5.8)			
4°	11 (9–18)		3 (1.3–6)		3 (1–4.8)			
**Regular exercise**								
Yes	11 (7–16)	0.045					6 (6–8)	0.017
No	13 (7.8–20)						8 (6–11)	

M (IQR): Median (Interquartile range).

We also analyzed whether age and BMI were related to the psychological indices (Table 4). On the one hand, we found that age was significantly correlated with all the indices studied (*p*< 0.035). Specifically, in the global PGWBI and its 6 dimensions, the correlations were negative, with correlation coefficients < 3.5. Whereas, in the global GHQ-12 index and its 3 dimensions, the correlations were positive, with coefficients < 2.5. On the other hand, BMI results were similar, negative correlations for the global PGWBI and its 6 dimensions and positive correlations for the global GHQ-12 index and its 3 dimensions, with correlation coefficients < 2. However, statistically significant associations were only found with self-esteem dimension, global PGWBI, anxiety dimension, depressed mood dimension, and positive well-being dimension (*p*< 0.05, see Table 4). Additionally, we observed a strong correlation between the global PGWBI and GHQ-12 scores (*r* = -0.858, *p*< 0.001), indicating that students with lower psychological well-being also have poorer general mental health (see Supplementary Figure 1S).

**Table 4:** Correlation of GHQ-12 and PGWBI index dimensions with age and body mass index.

Dimension	Age		Body mass index	
	r	p	r	p
GHQ-12 Global	0.195	**<0.010**	0.095	0.149
Coping strategies	0.141	**0.031**	0.013	0.848
Self-esteem	0.174	**<0.010**	0.134	**0.041**
Stress	0.214	**<0.010**	0.103	0.115
PGWBI global	-0.279	**<0.001**	-0.158	**0.016**
Anxiety	-0.250	**<0.001**	-0.134	**0.040**
Depressed mood	-0.250	**<0.001**	-0.168	**0.010**
Positive Well-being	-0.253	**<0.001**	-0.165	**0.011**
Self-control	-0.233	**<0.001**	-0.088	0.181
General health	-0.210	**<0.010**	-0.092	0.162
Vitality	-0.323	**<0.001**	-0.153	0.162

We subsequently analyzed characteristics associated with unfavorable scores in global PGWBI and global GHQ-12 index. For PGWBI, the unfavorable cutoff was set at 69 points, which corresponds to the median of the total population; for GHQ-12, the cutoff was set at 12 points, as reported by (Rocha et al., 2011). Thus, we sought variables associated with scores ≤ 69 for global PGWBI and ≥ 12 for global GHQ-12. Variables with statistically significant results were used to develop two multivariate models (see Table 5, Table 6).

**Table 5:** Factors related to unfavorable scores of GHQ-12 (≥ 12 points). Multivariate analysis.

Variable	B	Wald	OR	95 % C.I.	*p*-value
Tobacco	1.68	11.13	5.37	2.00–14.44	<0.001
Fast food consumption	1.39	11.92	4.01	1.82–8.83	<0.001
< 7 h of sleep	1.03	11.50	2.80	1.55–5.09	<0.001
Third academic course	0.76	4.40	2.13	1.05–4.33	0.036
Studying and working	0.24	0.21	1.27	0.46–3.54	0.646
Age	0.01	0.05	1.01	0.95–1.07	0.828
Regular exercise	-0.05	0.03	0.95	0.50–1.79	0.872

OR: Odds Ratio.

C.I: Confidence Interval.

**Table 6:** Factors related to unfavorable scores of PGWBI (≤ 69 points). Multivariate analysis.

Variable	B	Wald	OR	95 % C.I.	*p*-value
Tobacco	1.76	11.20	5.81	2.07–16.30	<0.001
Fast food consumption	1.27	10.09	3.55	1.62–7.76	0.001
< 7 h of sleep	0.96	10.42	2.62	1.46–4.70	0.001
Studying and working	0.06	0.02	1.07	0.39–2.94	0.901
Regular exercise	0.03	0.01	1.03	0.55–1.93	0.938
Body mass Index	0.00	0.03	1.00	0.98–1.02	0.875
Age	-0.02	0.26	0.98	0.92–1.05	0.612

OR: Odds Ratio.

C.I: Confidence Interval.

We found that 49.4 % and 51.5 % of participants had unfavorable scores for PGWBI and GHQ-12, respectively. Additionally, the factors associated with poor mental health scores in both questionnaires were: frequent fast-food consumption (OR > 3.5, 95 % C.I. 1.46–8.84), tobacco consumption (OR > 5, 95 % C.I. 2–16.30), and sleep less than seven hours (OR > 2.5, 95 % C.I. 1.46–5.09). Tobacco use showed the strongest association, with smokers being five times more likely to present unfavorable scores compared to non-smokers (See Table 5, Table 6). Finally, we observed that third-year students were 2.13 times more likely to present unfavorable scores on the global GHQ-12 index compared to students in other academic years (OR 2.13, 95 % C.I. 1.051–4.331) see Table 5.

## Discussion

To the best of our knowledge, this is the first study to evaluate the general well-being and psychological health of nursing students in Galicia, examining their relationship with sociodemographic and lifestyle factors. Our findings reveal a high prevalence of psychological distress and poor general mental health, affecting 49.4 % and 51.5 % of students, respectively. Key factors associated to these outcomes include lifestyle habits and being in the third year of study, which together shape a distinct profile of students experiencing psychological distress.

Consistent with previous studies, nursing students in their final year experience lower psychological well-being compared to those in earlier years (Smith and Yang, 2017; Sonmez et al., 2023). Our findings align with this, revealing that being in the third year is associated with reduced vitality, decreased self-esteem, and higher stress levels, which are linked to chronic psychological distress. This phenomenon could be attributed to various factors related to the academic and emotional demands they face at this stage of their education.

The final years of nursing school often involve a demanding combination of academic workload, final exams, and intensive clinical placements. This situation generates significant pressure on students, as they must balance theoretical learning with practical application in real-world healthcare settings. Clinical placements, while critical for their future careers, are a major source of stress. In these environments, nursing students often encounter direct patient care involving suffering, illness, death, and pain for the first time, which can be emotionally exhausting (Mendes and Martino, 2020). Additionally, the final year is marked by anxiety about future professional prospects. Students begin to worry about job opportunities, labor market competition, and the possibility of not securing employment immediately after graduation (Labrague et al., 2018). These circumstances can negatively impact students' psychological well-being, amplify their insecurities and significantly increase their stress levels (Rella et al., 2009).

Regarding lifestyle factors, our findings align with other studies that report a lack of exercise, insufficient sleep, smoking, and an inadequate diet are associated with psychological distress among nursing students (Smith and Yang, 2017; Sonmez et al., 2023; Yamashita et al., 2012). Numerous studies have shown that the absence of regular physical activity is one of the primary factors exacerbating psychological distress (Hawker, 2012; Ramón-Arbués et al., 2023). In our study, a lack of regular physical activity was linked to poor psychological well-being, reduced vitality, and diminished coping strategies. Exercise is known to release endorphins, neurotransmitters that act as natural painkillers and mood elevators This biochemical process not only helps in alleviating physical discomfort but also plays a crucial role in improving mood, reducing stress, and combating symptoms of anxiety and depression (Schoenfeld and Swanson, 2021). Regular exercise, therefore, can significantly enhance overall psychological well-being by promoting a sense of happiness and relaxation.

Sleep is essential for both physical and mental recovery. According to the Consensus Statement of the American Academy of Sleep Medicine and Sleep Research Society, adults should sleep at least seven hours per night to promote optimal health (29). Sleeping less than the recommended amount can significantly affect psychological well-being. Insufficient sleep is linked to difficulties in emotional regulation and higher levels of anxiety and depressive symptoms. For nursing students, inadequate rest due to academic and clinical demands is a common issue. A recent study by Ravi, R.K., and Mohamed M.G. revealed that 73 % of nursing students reported poor sleep quality, and 55.3 % slept less than seven hours per night (Kundayi Ravi et al., 2024). These findings align with our study, where 54 % of participants reported sleeping less than seven hours, which was directly associated with poorer psychological well-being.

Another critical lifestyle factor is smoking. In the present study, tobacco use showed a significant relationship with unfavorable outcomes across all dimensions of psychological well-being and mental health assessed. This suggests that students with poor psychological well-being may turn to smoking as a coping mechanism to alleviate emotional symptoms associated with stress or negative moods. This finding is consistent with a study conducted on 400 nursing students, where 50.9 % reported having started smoking in response to stress, sadness, or personal problems (Yiğitalp, 2015). It is important to note that, while smoking is sometimes perceived as a way to relieve stress, it has long-term detrimental effects on mental health. Nicotine may provide temporary relaxation but contributes to physical and emotional dependence, increasing anxiety levels and negatively affecting psychological well-being (Le Foll et al., 2022).

Regarding eating habits, it is well established that a balanced diet, including essential nutrients, provides the energy required for optimal physical and mental performance (Stanton et al., 2021). In our study, a diet primarily based on fast food was associated with unfavorable results across all evaluated dimensions. These findings are similar to those of a study conducted in northern Spain, where dietary fat intake was associated with anxiety among nursing students (Iglesias López et al., 2023). Frequent consumption of fast food among university students is often driven by multiple factors. This behavior is commonly attributed to poor time management, where students feel overwhelmed by their academic responsibilities and opt for quick, convenient, yet unhealthy food choices (Reuter et al., 2021). Additionally, it has been reported that some individuals turn to fast food as a source of temporary comfort and well-being. López Olivares et al. (2020) highlight that the consumption of hypercaloric foods can serve as a coping mechanism for anxiety and psychological distress (López-Olivares et al., 2020).

This combination of unhealthy habits may create a harmful cycle in which physical and psychological well-being progressively deteriorate (Sapranaviciute-Zabazlajeva et al., 2022). The findings of this study underscore the importance of implementing strategies aimed at improving lifestyle choices, optimizing time management, and strengthening stress-coping mechanisms. This is particularly relevant for nursing students, who, due to the demands of their training, represent a particularly vulnerable population.

### Recommendations

In many cases, nursing students lack adequate support and effective strategies to manage stress. The academic workload, clinical placements, and constant exposure to challenging situations in healthcare settings demand solid tools for managing anxiety and emotional tension. The lack of mental health resources, both at the individual and institutional levels, exacerbates this situation, increasing the risk of psychological distress (Chover-Sierra et al., 2024; Ramón-Arbués et al., 2023b; Smith and Yang, 2017).

Institutional support is a key factor in this context. Many universities and nursing training centers do not provide sufficient resources to help students cope with the stress inherent to the academic program. The stigma surrounding mental health in some educational settings may discourage students from seeking help, out of fear of being perceived as less competent or fragile (Abdelmonaem et al., 2024; Aloufi et al., 2021). For this reason, it is crucial to develop programs that offer psychological and emotional support while promoting self-care.

In this framework, several interventions have proven highly effective. For instance, mindfulness has been shown to help students develop greater awareness of their emotions and thoughts, allowing them to respond to stressful situations more calmly and reflectively instead of reacting impulsively. It also fosters an attitude of acceptance toward challenging experiences (Worsley et al., 2022), which is particularly useful in an emotionally demanding field like nursing. Additionally, cognitive-behavioral therapy (CBT) has been reported as a highly effective tool for university students, teaching them to identify and modify negative or distorted thought patterns that can exacerbate stress and anxiety (Benjet et al., 2023). In nursing, where self-imposed pressure and fear of making mistakes are common, learning to challenge harmful self-sabotaging thoughts could significantly reduce psychological distress.

Mind-body exercises, such as meditation, yoga, and relaxation and breathing techniques, have also been found effective in reducing stress, anxiety, and depressive symptoms in nursing students (Ji et al., 2024; Worsley et al., 2022).

Based on our findings, we also consider it necessary to promote campaigns on healthy lifestyle habits, including adequate nutrition, regular physical activity, and proper sleep hygiene. These campaigns should not only aim to inform but also raise awareness about the importance of adopting lifestyles that contribute to improved physical and mental health. Moreover, they should incorporate workshops and activities that actively involve students, providing them with practical tools to integrate these habits into their daily lives, both as current students and future healthcare professionals.

### Advantages and limitations

The primary advantage of this study is that, to our knowledge, it represents the first formal investigation in Galicia to comprehensively assess the degree of general well-being and psychological health among nursing students across all academic years, analyzing their relationship with sociodemographic and lifestyle factors. Furthermore, the main instruments used (PGWBI and GHQ-12) are validated for the Spanish population, ensuring the reliability of the results and enabling comparison with other research.

One of the major limitations of this study is its cross-sectional design, which does not allow for causal inferences. Thus, the conclusions are based on the relationships identified between the study factors, whose causal origins should be explored under more rigorous conditions suitable for such purposes. However, this design has allowed us to obtain an overview of the current situation and approach the underlying causes of psychological well-being and distress in nursing students.

It is also important to note that the generalizability of the results of this study may be limited due to the inclusion of nursing students from a single university in a specific region of southern Galicia. Consequently, these findings should be interpreted with caution, as they might not reflect the realities of students in other geographical areas or educational environment. The specific academic, social, and cultural context of this population may influence the observed associations between lifestyle factors and psychological well-being. For this reason, it is important that future studies explore whether similar patterns are observed in diverse populations and under different contextual conditions.

Another limitation is the lack of validated instruments to measure lifestyle-related factors. Questionnaires in this area tend to be lengthy, and our intention was to keep the survey concise to avoid losing student engagement. However, to mitigate the limitation of not having a validated instrument to measure lifestyle, we designed specific questions about activities and healthy lifestyle habits, grounded in data from previous studies (Breslow and Enstrom, 1980; Leyton et al., 2018; Reuter et al., 2021; Rocha et al., 2011.).

## Conclusions

This study has revealed a high prevalence of psychological distress and mental health issues among nursing students. It has also identified that factors such as lifestyle and being in the third year of study are closely related to these unfavorable outcomes, highlighting that lifestyle habits are potentially modifiable factors.

We believe these findings underscore the need for university authorities to implement interventions aimed at providing psychological support to students, along with preventive strategies in the field of mental health. It is important to remember that nursing students play an essential role in the healthcare system, as they are the future healthcare professionals. Therefore, fostering their psychological well-being from the beginning of their training by equipping them with tools to face the physical and emotional challenges of the profession is a crucial measure to prevent psychological distress in those who will become the pillars of the healthcare system in the future.

## Funding

This research did not receive any specific grant from funding agencies in the public, commercial, or not-for-profit sectors.

## CRediT authorship contribution statement

**Beatriz Calderón-Cruz:** Writing – review & editing, Writing – original draft, Visualization, Validation, Supervision, Resources, Project administration, Methodology, Investigation, Formal analysis, Conceptualization. **Uxía García-Sánchez:** Validation, Resources, Methodology, Investigation, Conceptualization. **Jacinto Luis González-Oya:** Writing – review & editing, Resources. **Ángela Prieto-Campo:** Writing – review & editing, Writing – original draft, Visualization, Validation, Supervision.

## Declaration of competing interest

The authors declare that they have no known competing financial interests or personal relationships that could have appeared to influence the work reported in this paper.

## Appendix Supplementary materials

Image, image 1

## References

[bib0001] Abdelmonaem Y.M.M., Osman M.A., Karim N.A.H.A. (2024). Mental health stigma and internship nursing students’ attitudes toward seeking professional psychological help: a cross-sectional study. BMC Nurs..

[bib0002] Almansoof A.S., Masuadi E., Al-Muallem A., Agha S. (2024). Prevalence of psychological distress among health sciences students: a systematic review and meta-analysis. Qual. Quant..

[bib0003] Alonso J., Mortier P., Auerbach R.P., Bruffaerts R., Vilagut G., Cuijpers P., Demyttenaere K., Ebert D.D., Ennis E., Gutiérrez-García R.A., Green J.G., Hasking P., Lochner C., Nock M.K., Pinder-Amaker S., Sampson N.A., Zaslavsky A.M., Kessler R.C., Boyes M., Kiekens G., Baumeister H., Kaehlke F., Berking M., Ramírez A.A., Borges G., Díaz A.C., Durán M.S., González R.G., de la Torre A.E.H., Martínez K.I.M., Medina-Mora M.E., Zarazúa H.M., Tarango G.P., Berbena M.A.Z., O’Neill S., Bjourson T., Roos J., Taljaard L., Saal W., Stein D., Almenara J., Ballester L., Barbaglia G., Blasco M.J., Castellví P., Cebrià A.I., Echeburúa E., Gabilondo A., García-Forero C., Iruin Á., Lagares C., Miranda-Mendizábal A., Parès-Badell O., Pérez-Vázquez M.T., Piqueras J.A., Roca M., Rodríguez-Marín J., Gili M., Soto-Sanz V. (2018). Severe role impairment associated with mental disorders: results of the WHO World Mental Health Surveys International College Student Project. Depress. Anxiety.

[bib0004] Aloufi M.A., Jarden R.J., Gerdtz M.F., Kapp S. (2021). Reducing stress, anxiety and depression in undergraduate nursing students: systematic review. Nurse Educ. Today.

[bib0005] Alshammari T.K., Al Harby N., Al Juffali L., Antonia Toto G., Limone P. (2022). Factors that predispose undergraduates to mental issues: a cumulative literature review for future research perspectives. Front. Public Health.

[bib0006] Arias-De la Torre J., Fernández-Villa T., Molina A.J., Amezcua-Prieto C., Mateos R., Cancela J.M., Delgado-Rodríguez M., Ortíz-Moncada R., Alguacil J., Redondo S., Gómez-Acebo I., Morales-Suárez-Varela M., Abellán G.B., Mejías E.J., Valero L.F., Ayán C., Vilorio-Marqués L., Olmedo-Requena R., Martín V. (2019). Psychological distress, family support and employment status in first-year university students in Spain. Int. J. Environ. Res. Public Health.

[bib0007] Badia X., Gutiérrez F., Wiklund I., Alonso J. (1996). Validity and reliability of the Spanish version of the Psychological General Well-Being Index. Qual. Life Res..

[bib0008] Benjet C., Zainal N.H., Albor Y., Alvis-Barranco L., Carrasco-Tapias N., Contreras-Ibáñez C.C., Cudris-Torres L., De La Peña F.R., González N., Guerrero-López J.B., Gutierrez-Garcia R.A., Jiménez-Peréz A.L., Medina-Mora M.E., Patiño P., Cuijpers P., Gildea S.M., Kazdin A.E., Kennedy C.J., Luedtke A., Sampson N.A., Petukhova M.V., Kessler R.C. (2023). A precision treatment model for internet-delivered cognitive behavioral therapy for anxiety and depression among university students: a secondary analysis of a randomized clinical trial. JAMA Psychiatry.

[bib0009] BOE-A-2008-12388 Orden CIN/2134/2008 (2008). https://www.boe.es/diario_boe/txt.php?id=BOE-A-2008-12388.

[bib0010] Bolton D. (2023). A revitalized biopsychosocial model: core theory, research paradigms, and clinical implications. Psychol. Med..

[bib0011] Breslow L., Enstrom J.E. (1980). Persistence of health habits and their relationship to mortality. Prev. Med..

[bib0012] Campbell F., Blank L., Cantrell A., Baxter S., Blackmore C., Dixon J., Goyder E. (2022). Factors that influence mental health of university and college students in the UK: a systematic review. BMC Public Health.

[bib0013] Chover-Sierra E., Saus-Ortega C., Labrague L.J. (2024). Umbrella review: stress levels, sources of stress, and coping mechanisms among student nurses. Nurs. Rep..

[bib0014] Estudios - Escuela Universitaria de Enfermeria Meixoeiro [WWW Document], n.d. URL https://direcceuemeixoeiro.webs.uvigo.es/estudios/(Accessed 8 July 2025).

[bib0015] Hawker C.L. (2012). Physical activity and mental well-being in student nurses. Nurse Educ. Today.

[bib0016] Iglesias López M.T., Marchena-Giráldez C.A., Bernabéu-Brotons E. (2023). Nutrient intake, alcohol consumption, emotional eating and anxiety in women nursing students. Heliyon.

[bib0017] Ji X., Guo X., Soh K.L., Japar S., He L. (2024). Effectiveness of stress management interventions for nursing students: a systematic review and meta-analysis. Nurs. Health Sci..

[bib0018] Kundayi Ravi R., Gamal Mohamed M., Professor A., Al Khiamah R. (2024). Sleep quality and influencing factors among nursing students: a cross-sectional survey from the United Arab Emirates. Nurs. Midwif. Stud..

[bib0019] Labrague L.J., McEnroe-Petitte D.M., De Los Santos J.A.A., Edet O.B. (2018). Examining stress perceptions and coping strategies among Saudi nursing students: a systematic review. Nurse Educ. Today.

[bib0020] Le Foll B., Piper M.E., Fowler C.D., Tonstad S., Bierut L., Lu L., Jha P., Hall W.D. (2022). Tobacco and nicotine use. Nat. Rev. Dis. Prim..

[bib0021] Leyton M., Lobato S., Batista M., Aspano M.I., Jiménez R. (2018). Validación del cuestionario de estilo de vida saludable (evs) en una población Española. Rev. Iberoam. Psicol. Ejerc. Deporte.

[bib0022] Límites de Consumo de Bajo Riesgo de Alcohol. [WWW Document], 2020. URL https://www.sanidad.gob.es/areas/promocionPrevencion/alcohol/documentosTecnicos/docs/Limites_Consumo_Bajo_Riesgo_Alcohol_Actualizacion.pdf (Accessed 15 July 2025).

[bib0023] López-Olivares M., Mohatar-Barba M., Fernández-Gómez E., Enrique-Mirón C. (2020). Mediterranean diet and the emotional well-being of students of the campus of Melilla (University of Granada). Nutrients.

[bib0024] Mendes S.S., Martino M.M.F.De (2020). Stress factors of nursing students in their final year. Rev. Esc. Enferm. USP..

[bib0025] Palenzuela-Luis N., Duarte-Clíments G., Gómez-Salgado J., Rodríguez-Gómez J.Á., Sánchez-Gómez M.B. (2023). Comparison between self-concept, self-perception, physical exercise and lifestyle variations from 1st to 4th grade students of nursing in Tenerife, Spain. Medicine.

[bib0026] Purtle J., Nelson K.L., Counts N.Z., Yudell M. (2020). Population-based approaches to mental health: history, strategies, and evidence. Annu. Rev. Public Health.

[bib0027] Ramón-Arbués E., Granada-López J.M., Satústegui-Dordá P.J., Echániz-Serrano E., Sagarra-Romero L., Antón-Solanas I. (2023). Psychological distress in nursing students: relationship with screen time, diet and physical activity. Rev. Lat. Am. Enfermagem.

[bib0028] Rella S., Winwood P.C., Lushington K. (2009). When does nursing burnout begin? An investigation of the fatigue experience of Australian nursing students. J. Nurs. Manage.

[bib0029] Reuter P.R., Forster B.L., Brister S.R. (2021). The influence of eating habits on the academic performance of university students. J. Am. Coll. Health.

[bib0030] Reverté-Villarroya S., Ortega L., Raigal-Aran L., Sauras-Colón E., Ricomà-Muntané R., Ballester-Ferrando D., Rascón-Hernán C., Botigué T., Lavedán A., González-Osorio L., Osorio-Spuler X., Burjalés-Martí M.D. (2021). Psychological well-being in nursing students: a multicentric, cross-sectional study. Int. J. Environ. Res. Public Health.

[bib0031] Rocha K.B., Pérez K., Rodríguez-Sanz M., Borrell C., Obiols JE. (2011). Propiedades psicométricas y valores normativos del General Health Questionnaire (GHQ-12) en población general española 1. Int. J. Clinic. Health Psychol..

[bib0032] Ruggeri K., Garcia-Garzon E., Maguire Á., Matz S., Huppert F.A. (2020). Well-being is more than happiness and life satisfaction: a multidimensional analysis of 21 countries. Health Qual. Life Outcom..

[bib0033] Sánchez Casado J.I., Benítez Sánchez E.I. (2021). Estudio de la salud mental en estudiantes universitarios de la rama sociosanitaria. Int. J. Develop. Educ. Psychol..

[bib0034] Sánchez López M.P., Dresch V. (2008). The 12-Item General Health Questionnaire (GHQ-12): reliability, external validity and factor structure in the Spanish population. Psicothema.

[bib0035] Sapranaviciute-Zabazlajeva L., Sileikiene L., Luksiene D., Tamosiunas A., Radisauskas R., Milvidaite I., Bobak M. (2022). Lifestyle factors and psychological well-being: 10-year follow-up study in Lithuanian urban population. BMC Public Health.

[bib0036] Schoenfeld T.J., Swanson C. (2021). A runner’s high for new neurons? Potential role for endorphins in exercise effects on adult neurogenesis. Biomolecules.

[bib0037] Smith G.D., Yang F. (2017). Stress, resilience and psychological well-being in Chinese undergraduate nursing students. Nurse Educ. Today.

[bib0038] Sonmez Y., Akdemir M., Meydanlioglu A., Aktekin M.R. (2023). Psychological distress, depression, and anxiety in nursing students: a longitudinal study. Healthcare.

[bib0039] Stanton R., Best T., Williams S., Vandelanotte C., Irwin C., Heidke P., Saito A., Rebar A.L., Dwyer T., Khalesi S. (2021). Associations between health behaviors and mental health in Australian nursing students. Nurse Educ. Pract..

[bib0040] Tiga-Loza D.C., de Pérez L.B.A., Ramírez-Cruz M.Á., de Diego Cordero R. (2024). Factors related to mental health problems in nursing students: a multicenter study. Rev. Cuid..

[bib0041] Tung Y.J., Lo K.K.H., Ho R.C.M., Tam W.S.W. (2018). Prevalence of depression among nursing students: a systematic review and meta-analysis. Nurse Educ. Today.

[bib0042] VizHub (2021). https://vizhub.healthdata.org/gbd-results/.

[bib0043] Worsley J.D., Pennington A., Corcoran R. (2022). Supporting mental health and wellbeing of university and college students: a systematic review of review-level evidence of interventions. PLoS ONE.

[bib0044] Yamashita K., Saito M., Takao T. (2012). Stress and coping styles in Japanese nursing students. Int. J. Nurs. Pract..

[bib0045] Yiğitalp G. (2015). Factors affecting smoking status of nursing students and their addiction levels. Turk. Thorac. J..

[bib0046] Zhou L., Chankoson T., Wu Y.M., Cai E.L. (2023). Thriving psychological well-being in undergraduate nursing student: a grounded theory study with the life grid approach. BMC Nurs..

[bib0047] Zhou W., Yu Y., Yang M., Chen L., Xiao S. (2018). Policy development and challenges of global mental health: a systematic review of published studies of national-level mental health policies. BMC Psychiatry.

