# Supplementary figures and images for: Factors related to well-being and psychological health in nursing students: A cross-sectional survey
Source: Int J Nurs Stud Adv. 2025 Sep 10;9:100421. doi: 10.1016/j.ijnsa.2025.100421 (PMC12495231; doi:10.1016/j.ijnsa.2025.100421)

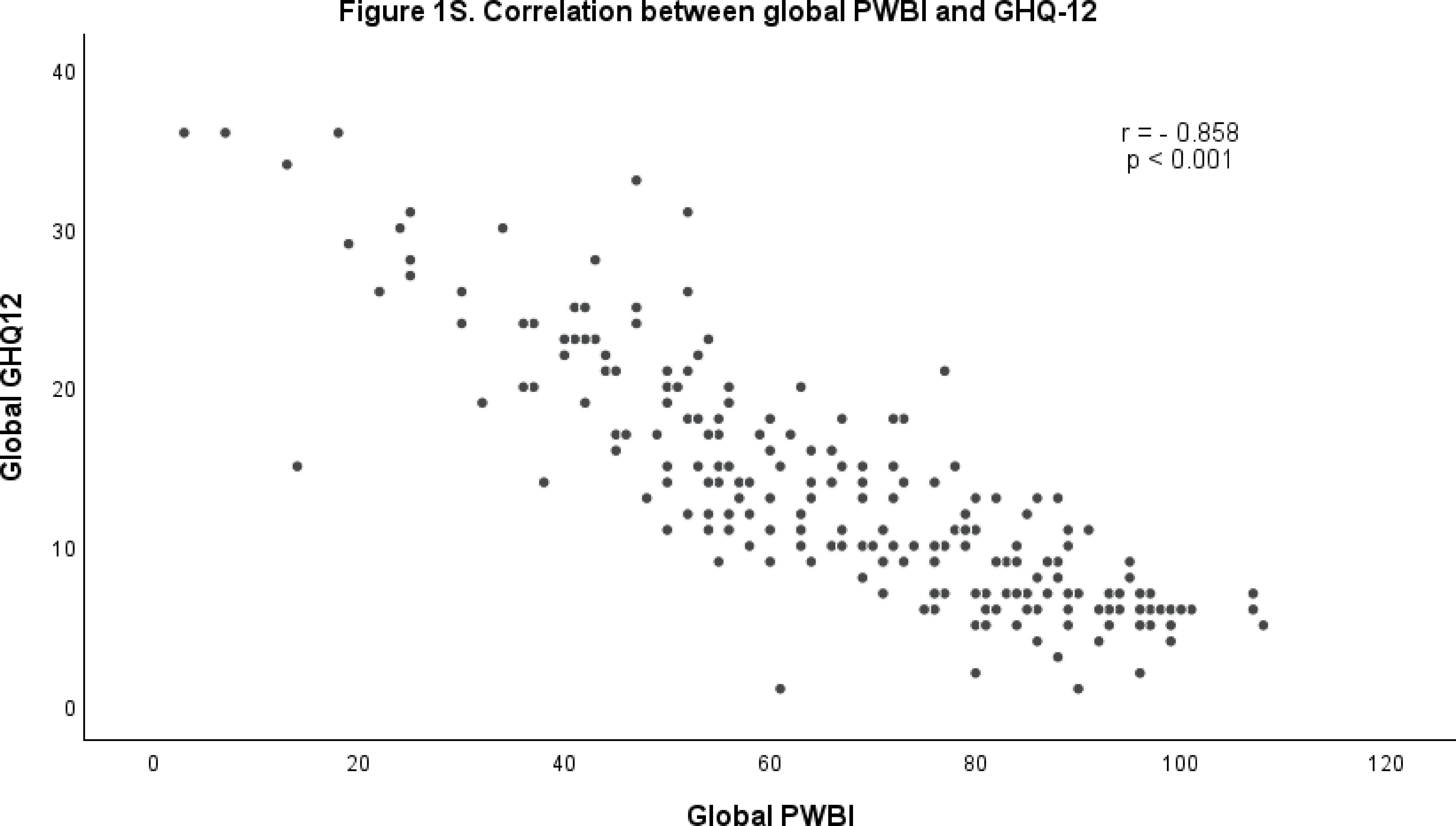

Supplement: Supplementary file 1 [file mmc1.jpg]
